# Crystal structure of 2,2′-bi­pyridine-1,1′-diium tetra­chlorido­zincate

**DOI:** 10.1107/S2056989015003175

**Published:** 2015-02-21

**Authors:** Jeyaraman Govindaraj, Subramani Thirumurugan, Antoni Samy Clara, Krishnamoorthy Anbalagan, Arunachalathevar SubbiahPandi

**Affiliations:** a Department of Physics, Pachaiyappa’s College for Men, Kanchipuram 631 501, India; b Department of Chemistry, Pondicherry University, Pondicherry 605 014, India; c Department of Physics, Presidency College (Autonomous), Chennai 600 005, India

**Keywords:** crystal structure, 2,2′-bi­pyridine-1,1′-diium, tetra­chlorido­zincate, hydrogen bonding

## Abstract

In the crystal structure of the title salt, (C_10_H_10_N_2_)[ZnCl_4_], the bi­pyridine­diium dication is not planar, with a dihedral angle of 37.21 (9)° between the planes of the two pyridine rings. In the crystal, the slightly distorted [ZnCl_4_]^2−^ anions are packed into rods parallel to [001], with the organic cations arranged in corrugated layers parallel to (100). Cations and anions are linked through N—H⋯Cl hydrogen bonds, forming chains parallel to [20-1]. Additional C—H⋯Cl inter­actions consolidate the crystal packing.

## Related literature   

For the crystal structure of 4,4′-bi­pyridine-1,1′-diium tetra­chlorido­zincate, see: Gillon *et al.* (2000 ▸). For other bi­pyridine derivatives with a [ZnCl_4_]^2−^ counter-anion, see: Rice *et al.* (2002 ▸).
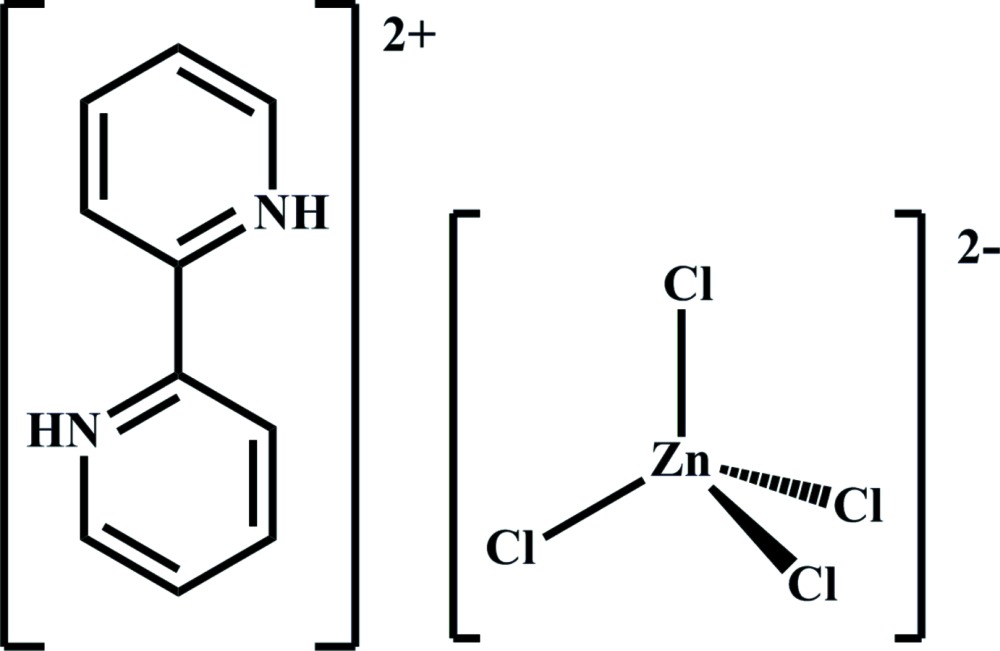


## Experimental   

### Crystal data  

(C_10_H_10_N_2_)[ZnCl_4_]
*M* *_r_* = 365.39
Monoclinic, 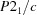

*a* = 7.1059 (4) Å
*b* = 13.6075 (6) Å
*c* = 14.2631 (7) Å
β = 100.816 (5)°
*V* = 1354.65 (12) Å^3^
*Z* = 4
Mo *K*α radiation
μ = 2.58 mm^−1^
*T* = 293 K
0.25 × 0.20 × 0.18 mm

### Data collection  

Oxford Diffraction Xcalibur diffractometer with an Eos detector
Absorption correction: multi-scan (*CrysAlis PRO*; Oxford Diffraction, 2009 ▸) *T*_min_ = 0.565, *T*_max_ = 0.654
7435 measured reflections
3115 independent reflections
2717 reflections with *I* > 2σ(*I*)
*R*_int_ = 0.024

### Refinement  

*R*[*F* ^2^ > 2σ(*F* ^2^)] = 0.025
*wR*(*F* ^2^) = 0.056
*S* = 1.05
3115 reflections
154 parameters
H-atom parameters constrained
Δρ_max_ = 0.49 e Å^−3^
Δρ_min_ = −0.51 e Å^−3^

### 

Data collection: *CrysAlis CCD* (Oxford Diffraction, 2009 ▸); cell refinement: *CrysAlis RED* (Oxford Diffraction, 2009 ▸); data reduction: *CrysAlis RED*; program(s) used to solve structure: *SHELXS97* (Sheldrick, 2008 ▸); program(s) used to refine structure: *SHELXL97* (Sheldrick, 2008 ▸); molecular graphics: *ORTEP-3 for Windows* (Farrugia, 2012 ▸); software used to prepare material for publication: *SHELXL97* and *PLATON* (Spek, 2009 ▸).

## Supplementary Material

Crystal structure: contains datablock(s) global, I. DOI: 10.1107/S2056989015003175/wm5125sup1.cif

Structure factors: contains datablock(s) I. DOI: 10.1107/S2056989015003175/wm5125Isup2.hkl

**Fig. 1.**
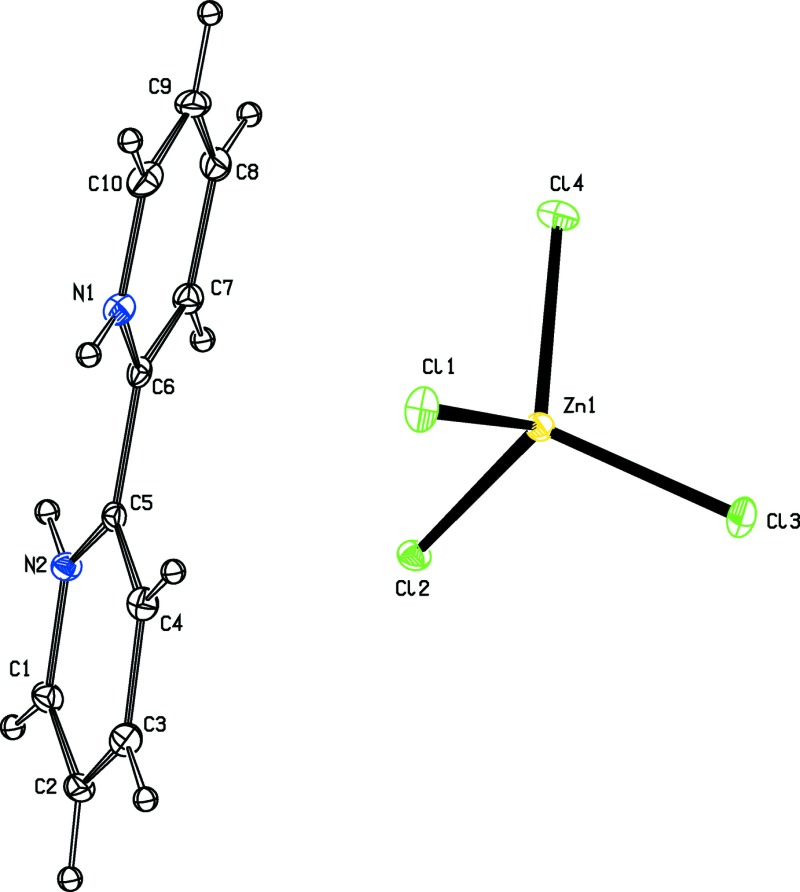
The molecular components of the title salt with displacement ellipsoids drawn at the 30% probability level.

. DOI: 10.1107/S2056989015003175/wm5125fig1.tif
The mol­ecular components of the title salt with displacement ellipsoids drawn at the 30% probability level.

**Fig. 2.**
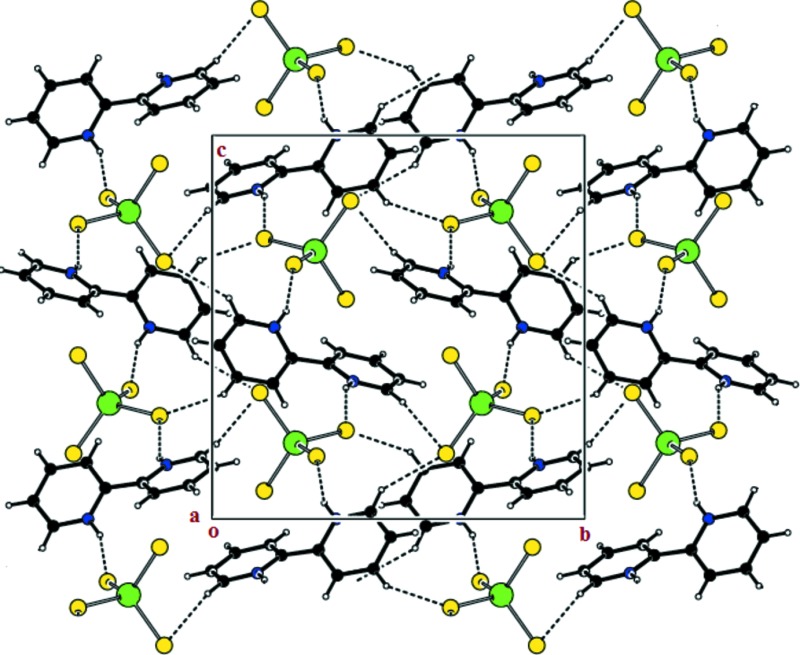
The crystal packing of the title compound viewed along [100].

. DOI: 10.1107/S2056989015003175/wm5125fig2.tif
The crystal packing of the title compound viewed along [100].

CCDC reference: 1049571

Additional supporting information:  crystallographic information; 3D view; checkCIF report

## Figures and Tables

**Table 1:** Hydrogen-bond geometry (, )

*D*H*A*	*D*H	H*A*	*D* *A*	*D*H*A*
N1H1*A*Cl4^i^	0.86	2.33	3.1058(15)	150
N2H2*A*Cl1^ii^	0.86	2.26	3.0693(15)	157
C1H1Cl2^iii^	0.93	2.74	3.4842(19)	137
C3H3Cl4^iv^	0.93	2.83	3.664(2)	150
C10H10Cl2^v^	0.93	2.67	3.570(2)	162

Symmetry codes: (i) 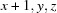; (ii) 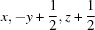; (iii) 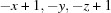; (iv) 
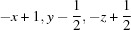
; (v) 
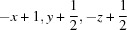
.

## supplementary crystallographic information

### S1. Experimental

Zinc chloride (136 mg, 1 mmol) was dissolved in 20 ml of water. To this solution was added dropwise 2,2'-bipyridine (156 mg, 1 mmol) in 20 ml of an EtOH/HCl mixture (1:9 *v*/*v*). The mixture was heated to 333 K for 2–3 hrs and allowed to stand until colorless crystals separated. The crystals were filtered and repeatedly recrystallized by using acidified water.

### S2. Refinement

N and C-bound H atoms were positioned geometrically (N—H = 0.86; C—H = 0.93 Å) and allowed to ride on their parent atoms, with *U*_iso_(H) = 1.2*U*_eq_(N, C).

### Crystal data

**Table:**

(C_10_H_10_N_2_)[ZnCl_4_]	*F*(000) = 728
*M**_r_* = 365.39	*D*_x_ = 1.791 Mg m^−^^3^
Monoclinic, *P*2_1_/*c*	Mo *K*α radiation, λ = 0.71073 Å
Hall symbol: -p 2ybc	Cell parameters from 2717 reflections
*a* = 7.1059 (4) Å	θ = 3.7–29.2°
*b* = 13.6075 (6) Å	µ = 2.58 mm^−^^1^
*c* = 14.2631 (7) Å	*T* = 293 K
β = 100.816 (5)°	Block, colourless
*V* = 1354.65 (12) Å^3^	0.25 × 0.20 × 0.18 mm
*Z* = 4	

### Data collection

**Table:**

Oxford Diffraction Xcalibur diffractometer with an Eos detector	3115 independent reflections
Radiation source: fine-focus sealed tube	2717 reflections with *I* > 2σ(*I*)
Graphite monochromator	*R*_int_ = 0.024
ω and φ scans	θ_max_ = 29.2°, θ_min_ = 3.7°
Absorption correction: multi-scan (*CrysAlis PRO*; Oxford Diffraction, 2009)	*h* = −9→9
*T*_min_ = 0.565, *T*_max_ = 0.654	*k* = −17→17
7435 measured reflections	*l* = −18→17

### Refinement

**Table:**

Refinement on *F*^2^	Primary atom site location: structure-invariant direct methods
Least-squares matrix: full	Secondary atom site location: difference Fourier map
*R*[*F*^2^ > 2σ(*F*^2^)] = 0.025	Hydrogen site location: inferred from neighbouring sites
*wR*(*F*^2^) = 0.056	H-atom parameters constrained
*S* = 1.05	*w* = 1/[σ^2^(*F*_o_^2^) + (0.0235*P*)^2^ + 0.1003*P*] where *P* = (*F*_o_^2^ + 2*F*_c_^2^)/3
3115 reflections	(Δ/σ)_max_ < 0.001
154 parameters	Δρ_max_ = 0.49 e Å^−^^3^
0 restraints	Δρ_min_ = −0.51 e Å^−^^3^

### Special details

**Table:**

Geometry. All e.s.d.'s (except the e.s.d. in the dihedral angle between two l.s. planes) are estimated using the full covariance matrix. The cell e.s.d.'s are taken into account individually in the estimation of e.s.d.'s in distances, angles and torsion angles; correlations between e.s.d.'s in cell parameters are only used when they are defined by crystal symmetry. An approximate (isotropic) treatment of cell e.s.d.'s is used for estimating e.s.d.'s involving l.s. planes.
Refinement. Refinement of *F*^2^ against ALL reflections. The weighted *R*-factor *wR* and goodness of fit *S* are based on *F*^2^, conventional *R*-factors *R* are based on *F*, with *F* set to zero for negative *F*^2^. The threshold expression of *F*^2^ > σ(*F*^2^) is used only for calculating *R*-factors(gt) *etc*. and is not relevant to the choice of reflections for refinement. *R*-factors based on *F*^2^ are statistically about twice as large as those based on *F*, and *R*- factors based on ALL data will be even larger.

### Fractional atomic coordinates and isotropic or equivalent isotropic displacement parameters (Å^2^)

**Table:**

	*x*	*y*	*z*	*U*_iso_*/*U*_eq_	
C1	0.6920 (3)	0.07397 (14)	0.51855 (13)	0.0181 (4)	
H1	0.6776	0.0453	0.5759	0.022*	
C2	0.7677 (3)	0.02053 (14)	0.45317 (14)	0.0200 (4)	
H2	0.8001	−0.0452	0.4643	0.024*	
C3	0.7947 (3)	0.06655 (14)	0.37010 (14)	0.0192 (4)	
H3	0.8492	0.0321	0.3256	0.023*	
C4	0.7409 (3)	0.16360 (14)	0.35321 (13)	0.0164 (4)	
H4	0.7598	0.1947	0.2977	0.020*	
C5	0.6590 (3)	0.21376 (13)	0.41919 (12)	0.0130 (4)	
C6	0.5805 (3)	0.31365 (14)	0.40546 (12)	0.0132 (4)	
C7	0.4136 (3)	0.34390 (14)	0.43280 (12)	0.0159 (4)	
H7	0.3465	0.3010	0.4652	0.019*	
C8	0.3461 (3)	0.43815 (15)	0.41188 (13)	0.0205 (4)	
H8	0.2330	0.4586	0.4297	0.025*	
C9	0.4473 (3)	0.50196 (14)	0.36437 (13)	0.0234 (4)	
H9	0.4041	0.5658	0.3506	0.028*	
C10	0.6122 (3)	0.46959 (14)	0.33786 (13)	0.0225 (4)	
H10	0.6811	0.5114	0.3052	0.027*	
N1	0.6743 (2)	0.37817 (11)	0.35883 (10)	0.0163 (3)	
H1A	0.7786	0.3595	0.3419	0.020*	
N2	0.6388 (2)	0.16722 (11)	0.50012 (10)	0.0147 (3)	
H2A	0.5896	0.1990	0.5417	0.018*	
Zn1	0.14622 (3)	0.224086 (15)	0.198218 (14)	0.01454 (7)	
Cl1	0.43041 (7)	0.28035 (4)	0.16341 (3)	0.02205 (12)	
Cl2	0.22538 (7)	0.12838 (3)	0.32961 (3)	0.01744 (10)	
Cl3	−0.01698 (8)	0.14035 (4)	0.07333 (4)	0.03026 (13)	
Cl4	−0.01829 (7)	0.35933 (3)	0.23116 (3)	0.02098 (11)	

### Atomic displacement parameters (Å^2^)

**Table:**

	*U*^11^	*U*^22^	*U*^33^	*U*^12^	*U*^13^	*U*^23^
C1	0.0177 (10)	0.0151 (9)	0.0199 (10)	−0.0006 (8)	−0.0003 (8)	0.0045 (7)
C2	0.0127 (10)	0.0140 (9)	0.0310 (11)	0.0012 (8)	−0.0017 (8)	−0.0010 (8)
C3	0.0121 (10)	0.0210 (10)	0.0250 (10)	0.0014 (8)	0.0050 (8)	−0.0068 (8)
C4	0.0136 (10)	0.0189 (10)	0.0174 (9)	−0.0007 (8)	0.0047 (7)	−0.0003 (7)
C5	0.0104 (9)	0.0139 (9)	0.0138 (9)	−0.0023 (7)	−0.0002 (7)	0.0003 (7)
C6	0.0160 (10)	0.0146 (9)	0.0081 (8)	−0.0026 (7)	0.0002 (7)	−0.0004 (7)
C7	0.0175 (10)	0.0158 (9)	0.0141 (9)	−0.0002 (8)	0.0027 (7)	−0.0009 (7)
C8	0.0213 (11)	0.0214 (10)	0.0172 (10)	0.0039 (8)	−0.0007 (8)	−0.0045 (8)
C9	0.0370 (13)	0.0130 (9)	0.0170 (10)	0.0050 (9)	−0.0032 (8)	0.0000 (8)
C10	0.0350 (12)	0.0164 (10)	0.0148 (10)	−0.0064 (9)	0.0015 (8)	0.0023 (7)
N1	0.0186 (9)	0.0158 (8)	0.0151 (8)	−0.0026 (7)	0.0044 (6)	−0.0005 (6)
N2	0.0171 (8)	0.0141 (8)	0.0132 (8)	0.0017 (6)	0.0035 (6)	−0.0009 (6)
Zn1	0.01358 (12)	0.01320 (12)	0.01649 (12)	−0.00066 (8)	0.00192 (8)	0.00128 (8)
Cl1	0.0188 (3)	0.0326 (3)	0.0162 (2)	−0.0072 (2)	0.00697 (18)	−0.00060 (19)
Cl2	0.0193 (2)	0.0146 (2)	0.0184 (2)	0.00016 (18)	0.00339 (17)	0.00387 (17)
Cl3	0.0305 (3)	0.0245 (3)	0.0295 (3)	−0.0056 (2)	−0.0106 (2)	−0.0036 (2)
Cl4	0.0207 (3)	0.0164 (2)	0.0278 (3)	0.00419 (19)	0.0097 (2)	0.00458 (19)

### Geometric parameters (Å, º)

**Table:**

C1—N2	1.336 (2)	C7—H7	0.9300
C1—C2	1.370 (3)	C8—C9	1.383 (3)
C1—H1	0.9300	C8—H8	0.9300
C2—C3	1.385 (3)	C9—C10	1.370 (3)
C2—H2	0.9300	C9—H9	0.9300
C3—C4	1.383 (3)	C10—N1	1.335 (2)
C3—H3	0.9300	C10—H10	0.9300
C4—C5	1.377 (2)	N1—H1A	0.8600
C4—H4	0.9300	N2—H2A	0.8600
C5—N2	1.348 (2)	Zn1—Cl3	2.2452 (5)
C5—C6	1.468 (2)	Zn1—Cl2	2.2647 (5)
C6—N1	1.350 (2)	Zn1—Cl4	2.2760 (5)
C6—C7	1.379 (2)	Zn1—Cl1	2.2994 (5)
C7—C8	1.382 (3)		
			
N2—C1—C2	120.20 (17)	C7—C8—C9	119.83 (18)
N2—C1—H1	119.9	C7—C8—H8	120.1
C2—C1—H1	119.9	C9—C8—H8	120.1
C1—C2—C3	118.53 (18)	C10—C9—C8	118.92 (18)
C1—C2—H2	120.7	C10—C9—H9	120.5
C3—C2—H2	120.7	C8—C9—H9	120.5
C4—C3—C2	120.15 (17)	N1—C10—C9	120.09 (18)
C4—C3—H3	119.9	N1—C10—H10	120.0
C2—C3—H3	119.9	C9—C10—H10	120.0
C5—C4—C3	119.53 (17)	C10—N1—C6	122.94 (17)
C5—C4—H4	120.2	C10—N1—H1A	118.5
C3—C4—H4	120.2	C6—N1—H1A	118.5
N2—C5—C4	118.61 (16)	C1—N2—C5	122.91 (16)
N2—C5—C6	116.77 (15)	C1—N2—H2A	118.5
C4—C5—C6	124.51 (16)	C5—N2—H2A	118.5
N1—C6—C7	118.37 (17)	Cl3—Zn1—Cl2	112.08 (2)
N1—C6—C5	117.22 (16)	Cl3—Zn1—Cl4	111.43 (2)
C7—C6—C5	124.33 (16)	Cl2—Zn1—Cl4	110.585 (18)
C6—C7—C8	119.84 (18)	Cl3—Zn1—Cl1	109.97 (2)
C6—C7—H7	120.1	Cl2—Zn1—Cl1	106.16 (2)
C8—C7—H7	120.1	Cl4—Zn1—Cl1	106.33 (2)
			
N2—C1—C2—C3	2.6 (3)	C5—C6—C7—C8	−176.39 (17)
C1—C2—C3—C4	−1.8 (3)	C6—C7—C8—C9	−0.6 (3)
C2—C3—C4—C5	−0.5 (3)	C7—C8—C9—C10	0.8 (3)
C3—C4—C5—N2	1.9 (3)	C8—C9—C10—N1	−0.7 (3)
C3—C4—C5—C6	−174.26 (18)	C9—C10—N1—C6	0.5 (3)
N2—C5—C6—N1	146.62 (17)	C7—C6—N1—C10	−0.2 (3)
C4—C5—C6—N1	−37.1 (3)	C5—C6—N1—C10	176.69 (16)
N2—C5—C6—C7	−36.7 (3)	C2—C1—N2—C5	−1.2 (3)
C4—C5—C6—C7	139.56 (19)	C4—C5—N2—C1	−1.1 (3)
N1—C6—C7—C8	0.3 (3)	C6—C5—N2—C1	175.37 (17)

### Hydrogen-bond geometry (Å, º)

**Table:**

*D*—H···*A*	*D*—H	H···*A*	*D*···*A*	*D*—H···*A*
N1—H1*A*···Cl4^i^	0.86	2.33	3.1058 (15)	150
N2—H2*A*···Cl1^ii^	0.86	2.26	3.0693 (15)	157
C1—H1···Cl2^iii^	0.93	2.74	3.4842 (19)	137
C3—H3···Cl4^iv^	0.93	2.83	3.664 (2)	150
C10—H10···Cl2^v^	0.93	2.67	3.570 (2)	162

Symmetry codes: (i) *x*+1, *y*, *z*; (ii) *x*, −*y*+1/2, *z*+1/2; (iii) −*x*+1, −*y*, −*z*+1; (iv) −*x*+1, *y*−1/2, −*z*+1/2; (v) −*x*+1, *y*+1/2, −*z*+1/2.
