# Supplementary figures and images for: Crystal structure of 2,2′-bi­pyridine-1,1′-diium tetra­chlorido­zincate
Source: Acta Crystallogr E Crystallogr Commun. 2015 Feb 21;71(Pt 3):m67–8. doi: 10.1107/S2056989015003175 (PMC4350760; doi:10.1107/S2056989015003175)

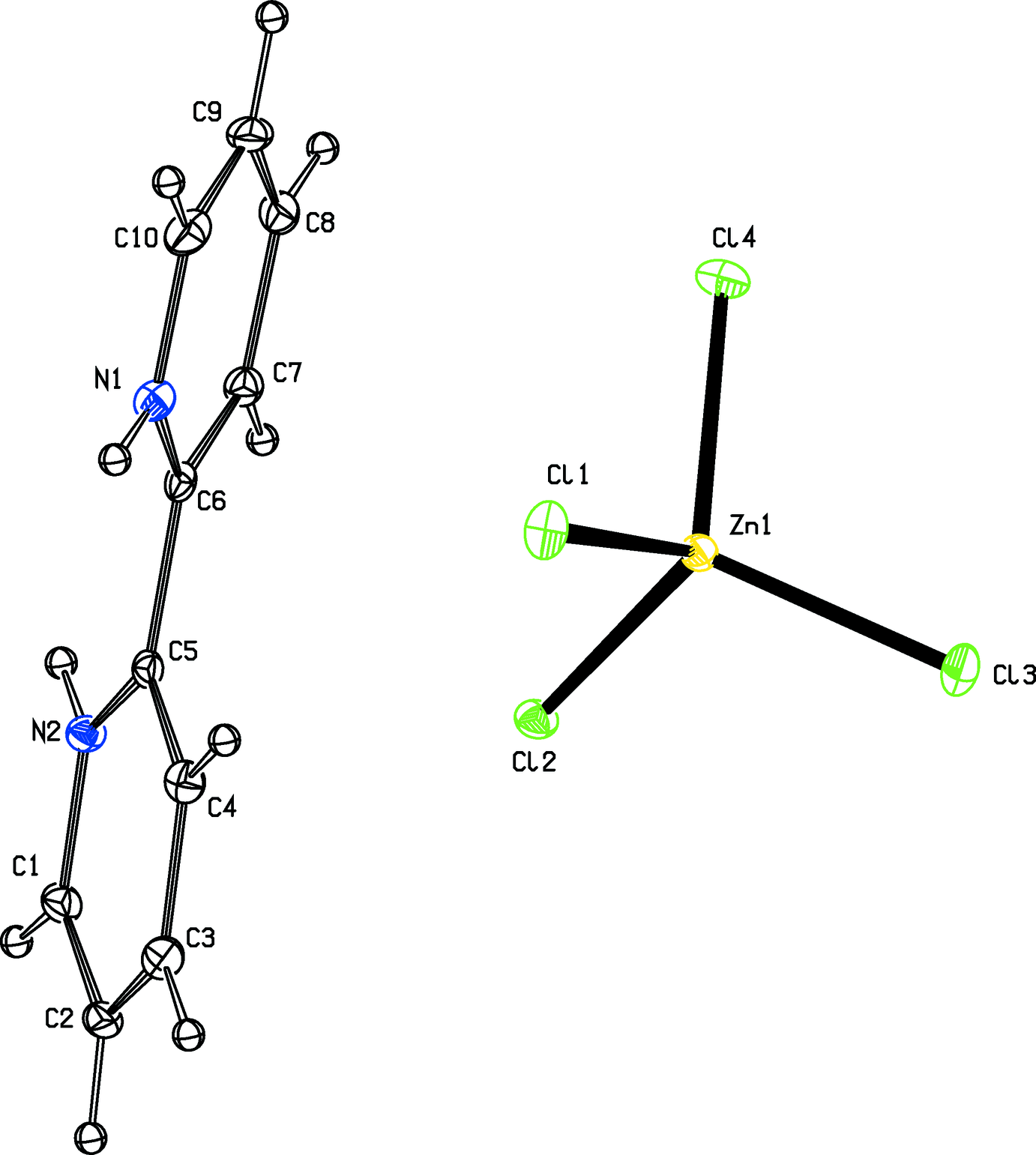

Supplement: Supplementary file 3 [file e-71-00m67-fig1.tif]

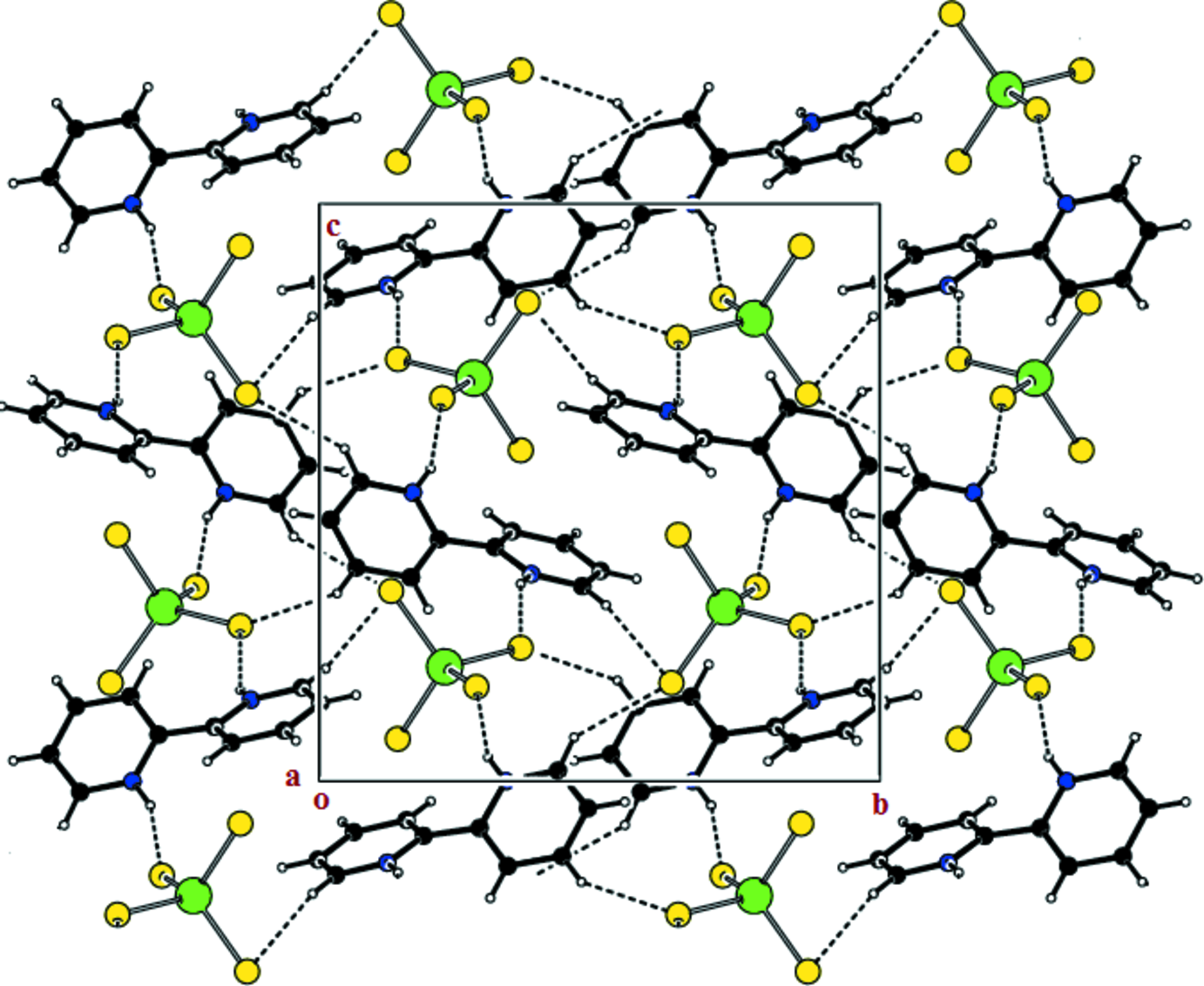

Supplement: Supplementary file 4 [file e-71-00m67-fig2.tif]
